# Blood Coagulation Testing Smartphone Platform Using Quartz Crystal Microbalance Dissipation Method

**DOI:** 10.3390/s18093073

**Published:** 2018-09-13

**Authors:** Jia Yao, Bin Feng, Zhiqi Zhang, Chuanyu Li, Wei Zhang, Zhen Guo, Heming Zhao, Lianqun Zhou

**Affiliations:** 1 CAS Key Laboratory of Bio-Medical Diagnostics, Suzhou Institute of Biomedical Engineering and Technology, Chinese Academy of Sciences, Suzhou 215163, China; yaojia@sibet.ac.cn (J.Y.); fengbincn@foxmail.com (B.F.); zhangzhiqi@sibet.ac.cn (Z.Z.); lichy@sibet.ac.cn (C.L.); zhangw@sibet.ac.cn (W.Z.); guozhen@sibet.ac.cn (Z.G.); 2 School of Electronic and Information Engineering, Soochow University, Suzhou 215006, China; hmzhao@suda.edu.cn; 3 University of Chinese Academy of Sciences, Beijing 100049, China

**Keywords:** blood coagulation, quartz crystal microbalance, dissipation, smartphone, point of care testing

## Abstract

Blood coagulation function monitoring is important for people who are receiving anticoagulation treatment and a portable device is needed by these patients for blood coagulation self-testing. In this paper, a novel smartphone based blood coagulation test platform was proposed. It was developed based on parylene-C coated quartz crystal microbalance (QCM) dissipation measuring and analysis. The parylene-C coating constructed a robust and adhesive surface for fibrin capturing. The dissipation factor was obtained by measuring the frequency response of the sensor. All measured data were sent to a smartphone via Bluetooth for dissipation calculation and blood coagulation results computation. Two major coagulation indexes, activated partial thromboplastin time (APTT) and prothrombin time (PT) were measured on this platform compared with results by a commercial hemostasis system in a clinical laboratory. The measurement results showed that the adjusted R-square (R^2^) value for APTT and PT measurements were 0.985 and 0.961 respectively. The QCM dissipation method for blood coagulation measurement was reliable and effective and the platform together with the QCM dissipation method was a promising solution for point of care blood coagulation testing.

## 1. Introduction

Blood coagulation tests are widely performed in the clinical laboratory since the coagulation indexes are significant to help the doctors to accurately assess the patients’ risk of excessive bleeding or clots developing. Blood coagulation function monitoring is important especially to those who are suffering from cardiovascular disease [1] or who are under anticoagulation treatment [2]. Patients who are taking warfarin or clopidogrel have to visit the physician’s office weekly to check their blood coagulation function to avoid hemorrhagic complications and achieve sufficient suppression of thrombosis. It will take much time to get the coagulation test result back and until then the new dose of anticoagulant medications can be discussed. A method or a device supporting patients’ blood coagulation function self-monitoring will help to improve this situation. It will make the blood coagulation function test to be an instant measurement and meet the demand of point-of-care testing [3].

Blood coagulation is propagated when tissue factor activates thrombin and then the resultant burst of thrombin converts fibrinogen to fibrin [4,5]. Activated partial thromboplastin time (APTT), prothrombin time (PT), thrombin time (TT) and fibrinogen (FIB) are referred to as the four major indexes in blood coagulation function assessment. Regular coagulation assays include clot-based tests [6,7], electrochemical measurements [8,9] and optical experiments [10]. Blood coagulation is a process in which liquid blood turns into gel clot, so it is a direct way of monitoring the viscoelastic change when blood coagulates [11], usually a coagulometer is needed [12,13,14].

QCM is a kind of acoustic sensors which has been widely used [15,16,17,18,19], frequency and dissipation are the two major properties which can be measured and traced. Frequency shift reflects the change of mass loaded on the sensor [20,21,22], while the dissipation factor gives viscoelastic information of the attached layer oscillating with QCM [23,24,25,26,27]. The shear waves QCM sensor employs will decay rapidly in the liquid environment, which limits the sensing depth of the sensor [28], so it is a vital task to construct an adhesive surface to capture fibrin onto the surface of the sensor. Several ways or biomaterials can be utilized to enhance the sensors’ capability of fibrin adhesion [29,30,31].

Studies on blood coagulation process using QCM sensor or resonators have been widely performed. Polyethylene coated 10 MHz QCM sensors were used to measure PT on the FidgeType FgT1 platform, frequency and dissipation were both provided [32]. Cantilever type resonators were packaged as cartridges which provided the ability to measure APTT and PT, an optical laser Doppler vibrometer was needed to measure the vibration amplitudes [33]. FBAR sensors were also used for PT measurement and only 1 μL sample was needed [34]. Although there were many research about blood coagulation, a portable, in vitro diagnostic device for blood coagulation function measurement was still absent.

**Figure 1:**
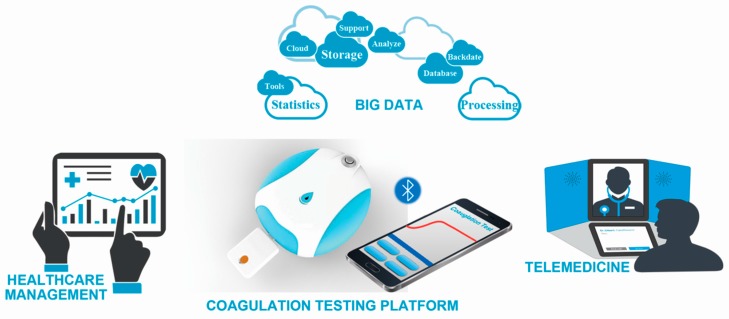
Coagulation testing platform and its functions.

In this paper, a QCM dissipation based approach for blood coagulation monitoring was proposed and a portable coagulation test platform was developed according to the QCM dissipation theory. The platform consisted of a test device, a smartphone and test chips. QCM sensors were packaged as disposable test chips for coagulation function assessments. The test device was used to perform frequency sweep and record the sensor’s response. All of the sampled data were sent to the smartphone via Bluetooth, where dissipation factor results and blood coagulation indexes were calculated employing the computation capability of the smartphone. With the coagulation test platform prepared, a system evaluation was performed to ensure that the performance of the platform would meet the demands of the blood coagulation function test. Then APTT and PT measurements were carried out in the clinical laboratory of a hospital. A commercial clinical coagulation analyzer adopting optical technique was used to do comparative experiments, the results of which were considered as standards. Coagulation test results could be stored in smartphone or data cloud for data backtracking or healthcare management and they could be sent to the physicians for consulting, the platform and its functions were shown in Figure 1.

## 2. Materials and Methods

### 2.1. Reagents and Equipment

APTT and PT test kits were purchased from Sun Biotech Co., Ltd., Shanghai, China. APTT test kits contained APTT activator (a mixture of cephalin and ellagic acid) and APTT starter (25 mM Calcium Chloride). Ethyl alcohol and hydrogen peroxide were bought from Sigma-Aldrich. Heparin sodium salt was purchased from Sinopharm Chemical Reagent Co., Ltd., Beijing, China. Parylene-C was purchased from Specialty Coating Systems, Indianapolis, IN, USA. 0.01 M phosphate buffer saline (PBS, PH7.2) was purchased from GE Healthcare Life Sciences. Dade Actin activated cephaloplastin and Thromborel-S reagents for the SYSMEX hemostasis system were purchased from SIEMENS Healthcare Diagnostics Products GmbH, Germany.

PDS 2010 chemical deposition system (Specialty Coating Systems, Indianapolis, IN, USA) was used to form parylene-C membrane on the QCM surface. Milli-Q academic filtration system (Merck KGaA, Darmstadt, Germany) was used to produce deionized water. An ultraviolet and visible spectrometer (UV/VIS/NIR Spectrometer Lambda 950, Perkin Elmer, Waltham, MA, USA) was used to perform APTT tests based on absorbance spectroscopy. A SYSMEX CS-5100 hemostasis system (Siemens Healthcare GmbH, Erlangen, Germany) was used to perform comparative tests of APTT and PT.

### 2.2. QCM Dissipation Based Coagulation Measurement Principles

QCM sensor is quite sensitive to the viscoelastic change of blood sample when blood coagulates and dissipation is the key factor and effective parameter to reflect this kind of transformation. QCM sensor can only probe the region very close to the interface since the shear wave decays rapidly in the liquid environment. The sensing depth of the QCM sensor can be described as [35]

(1) $$\;\delta ={\left(\frac{\eta }{\pi f_{0}\rho }\right)}^{\frac{1}{2}},\;$$

where δ, η,$\;f_{0}$, ρ represent the sensing depth of QCM, the viscosity of the liquid, the resonant frequency of QCM and the density of the liquid, respectively.

For a 10 MHz QCM sensor, assume that the viscosity of plasma η= 1.42 × 10^−3^ Pa·S and the density of plasma ρ =  1.03 × 10^3^ kg/m^3^, the sensing depth of the QCM sensor can be calculated as δ≈ 210 nm.

Adding coagulation reagents would initiate the clotting process, fibrinogen was finally enzyme catalyzed to fibrin and fibrin started to attach to the sensor’s surface. Since the sensing depth was limited to only about 210 nm, a rigid parylene-C film was coated on the surface of the electrode to form a uniform and robust interface for fibrin adhesion, a protein layer will be formed close to the parylene-C film. According to Kevin-Voigt model, the QCM sensor’s dissipation change can be described as [36]

(2) $$∆D\approx \frac{1}{\pi f_{0}\rho_{0}h_{0}}\left\{\frac{\eta }{\delta }+2h_{1}{\left(\frac{\eta }{\delta }\right)}^{2}\frac{\eta_{1}\omega }{\mu_{1}{}^{2}+\omega^{2}\eta_{1}{}^{2}}\right\},$$

where $f_{0}$,$\;\rho_{0}$ and $h_{0}$ are the resonant frequency, the density and the thickness of the sensor. $h_{1}$,$\;\mu_{1}$,$\;\eta_{1}$ represent the thickness, the shear elasticity and the viscosity of the protein layer. η is the viscosity of the plasma. δ is the penetration depth of the shear wave. ω is the angular frequency of the resonant oscillation.

**Figure 2:**
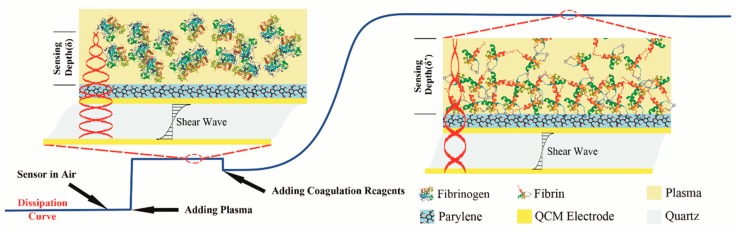
Quartz Crystal Microbalance (QCM) dissipation based coagulation measurement principles.

The QCM dissipation based coagulation process can be depicted as Figure 2.

### 2.3. Platform Electronic Design Principles

Many electronic circuits could be used for QCM sensors [37,38], in this paper the dissipation factor of a QCM sensor could be calculated as the reciprocal of the sensor quality factor (*Q*) and it was defined as [39]

(3) $$D=\frac{1}{Q}=\frac{BW}{f_{s}},$$

where:

*D*—The dissipation factor of the QCM sensor,
*BW*—3 dB bandwidth of the center frequency, in Hertz,
*f_s_*—The resonant frequency, in Hertz.

Although the dissipation factor of harmonic resonant frequency could be calculated similarly, only fundamental dissipation factor was considered and calculated in this paper.

**Figure 3:**
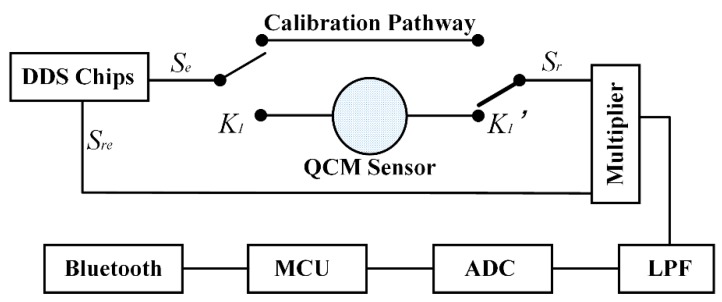
The principles of the test device design.

The bandwidth value (*BW*) and the resonant frequency value (*f_s_*) were both computed from the frequency response curve of the QCM sensor and the frequency response curve was obtained point by point. Referring to Figure 3, we followed below steps to obtain the response of a single frequency point (e.g., *f_n_*).

1. Switches *K*_1_ and *K*_1_’ were left unconnected to any pathway to start a dark noise calibration;
2. DDS chips were used to generate two sine wave signal *S_e_* and *S_re_* with the same frequency (*f_n_*) and the same phase (0°), the result of the multiplier was processed by low pass filter (LPF) and analog to digital converter (ADC), the voltage was recorded as $V_{dark-0}$;
3. Keep the signal *S_e_* unchanged and *S_re_* was generated with frequency $f_{n}$ and phase 90°, the voltage result was recorded as $V_{dark-90}$;
4. *K*_1_ and *K*_1_’ were switched to calibration pathway to start a single frequency zero-point calibration.
5. Repeat steps 2–3, voltage results were recorded as $V_{cali-0}$ and $V_{cali-90}$ respectively;
6. *K*_1_ and *K*_1_’ were switched to QCM pathway to start a single point frequency response measurement;
7. Repeat steps 2–3, voltage results were recorded as $V_{mea-0}$ and $V_{mea-90}$ respectively;

All the values were sent to the smartphone and the magnitude frequency response at the single frequency point *f_n_* could be calculated by

(4) $$Magnitude\left(f_{n}\right)=M_{mea}\left(f_{n}\right)-M_{cali}\left(f_{n}\right),$$

where

(5) $$M_{mea}\left(f_{n}\right)=20lg\left(\sqrt{{\left(V_{mea-0}-V_{dark-0}\right)}^{2}+{\left(V_{mea-90}-V_{dark-90}\right)}^{2}}\right),$$

(6) $$M_{cali}\left(f_{n}\right)=\;20lg\left(\sqrt{{\left(V_{cali-0}-V_{dark-0}\right)}^{2}+{\left(V_{cali-90}-V_{dark-90}\right)}^{2}}\right).$$

The center frequency of the QCM we used was around 10 MHz. We swept from 9.98 MHz to 10.02 MHz with 200 frequency points and then a third polynomial fitting was carried out to get the magnitude frequency response curve. *BW* and *f_n_* could be both determined from the curve and then the dissipation factor could be calculated according to Equation (1). With the real-time dissipation results calculated and tracked, APTT and PT results could be computed and monitored.

### 2.4. Coagulation Test Chip Design

**Figure 4:**
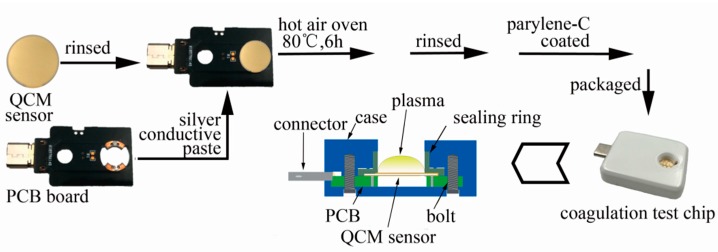
Coagulation test chip design approach.

Each QCM sensor was packaged as a coagulation test chip. The steps we took were shown in Figure 4. First, the QCM sensor was rinsed by ethyl alcohol, hydrogen peroxide and deionized water sequentially and dried by nitrogen. Second, the QCM sensor was bonded on a print circuit board (PCB) with silver conductive paste and the component was kept in a hot-air oven with temperature controlled at 80 °C for 6 h until the silver paste was all hardened. Finally, the sensor component was rinsed by ethyl alcohol, hydrogen peroxide and deionized water sequentially, dried by nitrogen.

Parylene-C coating had been verified as an impactful surface modification method when performing coagulation assessment. This would enhance the QCM sensor’s stability and robustness but the sensor’s quality and sensitivity would not be deteriorated much [40]. The previously assembled sensor component was put into the deposition chamber of the PDS2010 with the back side protected by adhesive silicone tape. First, 0.5 g parylene-C dimmer was put into the feeding chamber and a system vacuum was implemented until a pressure of 20 mTorr was reached. Then the temperature of the feeding chamber began to rise and the parylene-C dimmer in the feeding chamber began to vaporize at the temperature of 175 °C and began to pyrolyze at a temperature of 690 °C. At last, the gaseous parylene-C was allowed to diffusion into the deposition chamber and a thin layer of parylene-C film was formed on the surface of the QCM sensor. The thickness of the coated film was around 450 nm.

The parylene coated component was then packaged as a coagulation test chip. The top surface of the QCM sensor and the rubber sealing ring formed a chamber where the blood coagulation would take place. The chip was then connected to the test device by a USB type-c connector.

### 2.5. Dissipation Stability of the Test Device

**Figure 5:**
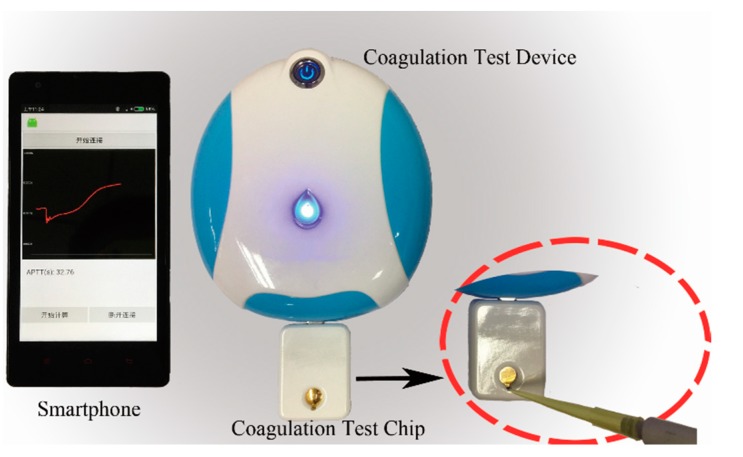
A photograph of the coagulation testing platform.

A photograph of the coagulation testing platform was shown in Figure 5 and a pipette was needed to load blood samples onto the sensor. An evaluation experiment was done in laboratory conditions to confirm the performance of the platform. The room temperature was air-conditioner controlled at 25 °C. Since a single coagulation test would be finished within 15 min, this required the device to have high short-term stability. We spent an hour to evaluate the dissipation stability of the device. First, a test chip with nothing in the chamber was connected to the test device and dissipation factor was recorded for half an hour, then 150 μL PBS solution was added into the chamber, ensuring that the top electrode was entirely covered and no bubbles were generated and another half an hour was spent on dissipation data recording.

**Figure 6:**
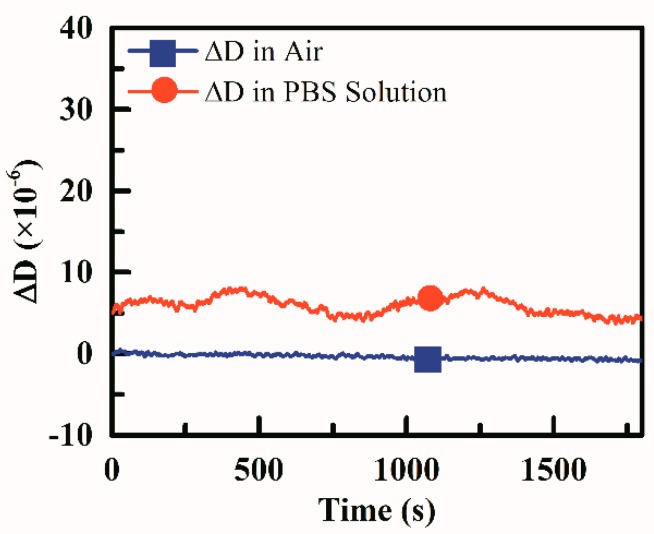
Dissipation stability performance of the platform.

The stability evaluation results were shown in Figure 6, Variations of 1 × 10^−6^ in the air and 2.5 × 10^−6^ in the PBS solution could be observed. This verified that the device was qualified for coagulation test since the dissipation change during coagulation was much more than 2.5 × 10^−6^.

### 2.6. APTT and PT Measurements Based on the Platform

All APTT and PT measurements were carried out in Suzhou Hospital Affiliated to Nanjing Medical University, Suzhou, China.

Sodium citrate 1:9 vacuum blood collection tubes were used to collect whole blood samples from healthy donors. Since the clinical hemostasis system required plasma samples, platelet poor plasma was also used in APTT and PT experiments for the convenience of results comparison. The tubes were placed in a centrifugal machine and each whole blood sample was separated into three layers after it was centrifuged at 3000 rpm for 15 min. The top layer of the sample was platelet-poor plasma we needed and pipettes were used to collect each plasma samples into different Eppendorf tubes for later measurements.

All measurements were carried out with the room temperature controlled at 25 °C. And before each measurement, a coagulation sensor chip was connected to the device until a stable dissipation baseline was obtained and the variation was about 2 × 10^−6^/min.

When performing QCM measurements, the whole working electrode should be covered by samples, or else signal fluctuations would be encountered. A minimum volume of 150 μL reaction system was needed when the QCM based coagulation test chip was used in our device to make sure the working electrode was all covered during the whole measurement. According to the requirement of plasma to reagent ratio, 50 μL APTT activator, 50 μL APTT starter and 50 μL plasma were used when performing APTT measurements and 75 μL PT reagent and 75 μL plasma were used.

For APTT measurements, 50 μL APTT activator was mixed with 50 μL plasma with a pipette for 3 min and then the mixture was loaded onto the QCM sensor, making sure that the whole top electrode was covered and no bubbles generated. Another 1 min was spent waiting for the dissipation signal to settle down. 50 μL APTT starter was then added into the mixture with a pipette, the step of which would induce the intrinsic coagulation process.

Normally, prothrombin time would be around 10 s, to enhance the resolution of the test results, prothrombin time was prolonged by reducing the PT reagents. First, 75 μL plasma was loaded onto the QCM sensor and a dissipation variation of 2 × 10^−6^/min should be reached before next step. Then 75 μL PT reagent was added into the chamber which induced the extrinsic coagulation process.

## 3. Results and Discussions

### 3.1. Dissipation Based Coagulation Measurement

There were two major parameters could be traced when using QCM sensors: frequency and dissipation. Frequency shift was proportional to the mass change according to the Sauerbrey equation for a rigid, thin, uniform film and the dissipation change should be less than 1 × 10^−6^ per 10 Hz frequency change [41,42]. While in the coagulation process the ratio of dissipation change to frequency change would go up to 2.5 × 10^−6^/10 Hz, as a result, the total adsorbed mass was easily underestimated [43] and the fluctuations might occur due to the non-uniform adhesion of fibrin onto the surface of the QCM electrode. When the dissipation factor was used, not only the resonant frequency but also the energy loss was taken into consideration. All these made the dissipation factor a more suitable parameter to be traced in coagulation testing and the viscoelasticity was the major change when the phase state of blood changed from liquid to gel. The increasing viscoelastic characteristic of the sample was directly expressed by the sensor’s dissipation factor shift.

### 3.2. Coagulation Indexes Results Determination

**Figure 7:**
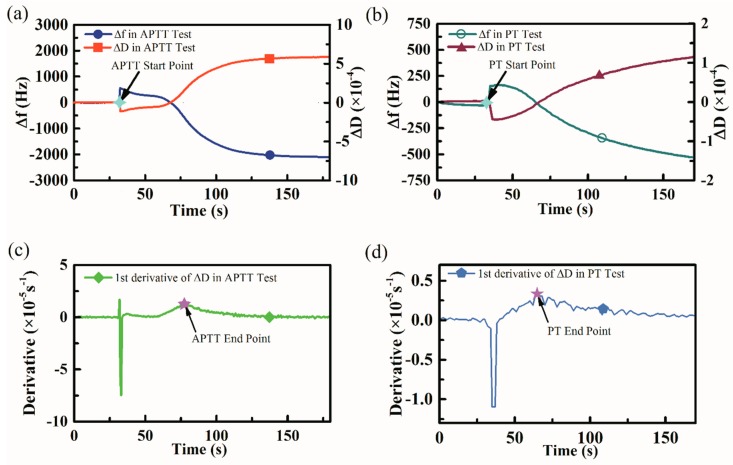
Typical frequency and dissipation signals during blood coagulation, all data were recorded by a smartphone. (**a**) Typical frequency and dissipation curves during activated partial thromboplastin time (APTT) measurements and the APTT start point was considered as the moment when APTT activator was added. (**b**) Typical frequency and dissipation curves during PT measurements and the PT start point was considered as the moment when PT reagent was added. (**c**) The first-order derivative of APTT dissipation curve and the peak point was regarded as the APTT end point. (**d**) The first-order of PT dissipation curve and the peak point was regarded as the PT end point.

The typical frequency and dissipation change curves during blood coagulation were shown in Figure 7. For APTT measurements, referring to Figure 7a, when the mixture of APTT activator and plasma was loaded onto the QCM sensor, the dissipation factor would increase dramatically and reach a stable baseline. The coagulation process was activated when the APTT starter was added into the mixture and this particular moment was considered as the APTT start point. The dissipation factor curve would fluctuate for a while because of the sample loading operation and the fibrin started to form and attach to the parylene-C membrane, the dissipation factor would gain slowly in this stage. Then the rapid increase of dissipation could be observed because the burst of thrombin catalyzed and accelerated the fibrin formation. At last, the dissipation would return to a stationary phase until the coagulation completely finished. A first-order derivative processing of the dissipation curve was performed referring to Figure 7c and the peak point of the derivative curve was considered as the APTT end point. The time elapsed between the APTT start point and APTT end point was regarded as the typical APTT value proportional to the true APTT value.

Similarly, Figure 7b showed the frequency, dissipation curve when performing PT measurements and the determination of the PT tests start time point. Figure 7d showed the first-order derivative curve of the dissipation curve in Figure 7b and the PT tests end point was determined as the peak point of the derivative curve.

### 3.3. APTT and PT Measurement Results

All coagulation experiments were carried out at a controlled temperature, although it was not 37 °C. This resulted in a prolongation of APTT and PT time [44]. Due to the consistent temperature condition, the prolongation was also consistent and could be compensated later.

**Figure 8:**
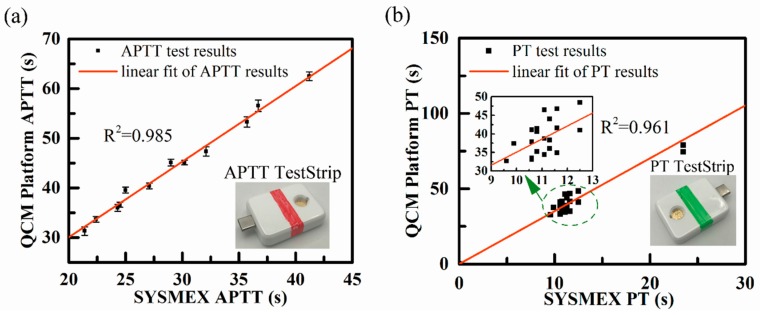
QCM coagulation testing platform results. (**a**) Comparison of twelve APTT measurements between the testing platform and CS-5100 hemostasis system. (**b**) Comparison of PT measurements between the testing platform and CS-5100 hemostasis system.

We carried out twelve APTT measurements using samples from twelve different healthy donors, with each sample tested for three times. The samples were also tested by CS-5100 hemostasis system in the clinical laboratory, the results of CS-5100 were considered as the standard APTT results. All the test results were shown in Figure 8a. A linear fitting was done based on these results with an R^2^ value of 0.985, which proved that the system had great test linearity.

PT measurements were carried out based on twenty-three experiments with different plasma samples and the results were shown in Figure 8b. SYSMEX hemostasis system showed that the PT results of the samples were within the range of 10.6 s to 23.5 s. Two of the samples had abnormal PT values, which could be distinguished from normal ones by our device. The results calculated by the coagulation test device owned linearity with an R^2^ value of 0.961.

### 3.4. Reducing the Sample Volume of Coagulation Measurements

**Figure 9:**
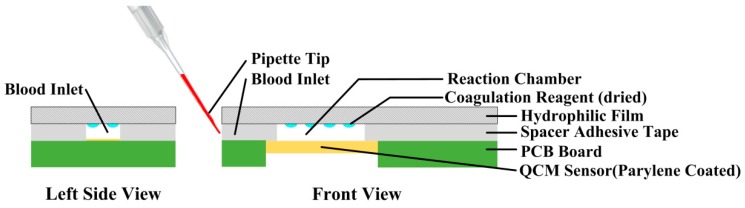
The structure of the further developed coagulation test chip to reduce the sample volume.

When performing coagulation tests, 75 μL plasma sample was required for PT measurements and 50 μL plasma sample was required for APTT measurements. To reduce the volume of blood sample when coagulation tests were conducted, the coagulation test chip was further developed as shown in Figure 9, the QCM sensor was mounted on PCB board with silver paste and a thin layer of parylene-C was coated on the QCM sensor as mentioned above.

Then a layer of spacer adhesive tape was used to paste onto the QCM sensor component, left sensor electrode and blood inlet area uncovered.

Coagulation reagents were dropped onto a hydrophilic film by a low volume fluid dispensing system and then dried in a hot-air oven. The film was then covered on the spacer, this would provide a reaction chamber which was about 6 μL in volume. Pipettes were used to load either plasma or whole blood into the inlet of the coagulation test chip and the samples would be guided to the reaction chamber utilizing the hydrophilic characteristic of the hydrophilic film.

**Figure 10:**
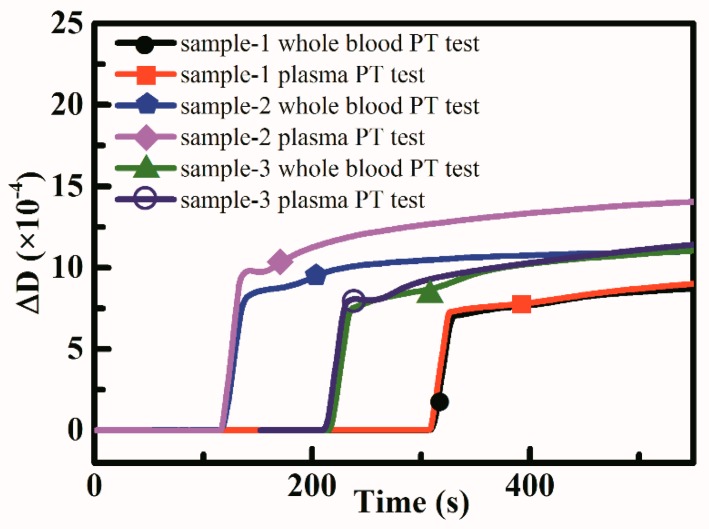
Three samples were used to perform PT measurements based on whole blood and plasma.

Three samples from healthy donors were used to perform whole blood based PT tests and plasma based PT tests. The QCM dissipation change curve result was shown in Figure 10 and the PT results calculated was shown in Table 1. Whole blood based PT tests showed close results compared to plasma based PT tests, which showed the possibility to measure coagulation function using whole blood.

## 4. Conclusions

In this paper, a novel portable, smartphone-based blood coagulation function test platform was introduced. Dissipation parameter of QCM sensor was more suitable than frequency to be traced in blood coagulation test since the layer formed on the QCM electrode was not rigid and it was a direct way to monitor the viscoelasticity change when blood coagulated. Parylene-C coating treatment made the QCM sensor more reliable and robust. A series of APTT and PT tests were performed by the device, the adjusted R-square (R^2^) values were 0.985 and 0.961 respectively. The results showed great reliability and linearity. With this device and platform, blood coagulation disorder patients will be able to achieve coagulation function self-testing or healthcare self-management. The platform could also be used for remote medical services or in hierarchical medical systems.

## Figures and Tables

**Table 1:** PT test results comparison (Mean ± SD were provided for QCM based test results).

Samples	Whole Blood Based PT Test Results (s)	Plasma Based PT Test Results (s)	Reference PT Test Results (s)
sample-1	72.1 ± 1.5	82.2 ± 5.4	10.8
sample-2	67.0 ± 6.4	77.1 ± 3.4	10.7
sample-3	85.3 ± 8.4	88.2 ± 10.8	10.9
